# Supplementary figures and images for: Generation of full-length circular RNA libraries for Oxford Nanopore long-read sequencing
Source: PLoS One. 2022 Sep 7;17(9):e0273253. doi: 10.1371/journal.pone.0273253 (PMC9451095; doi:10.1371/journal.pone.0273253)

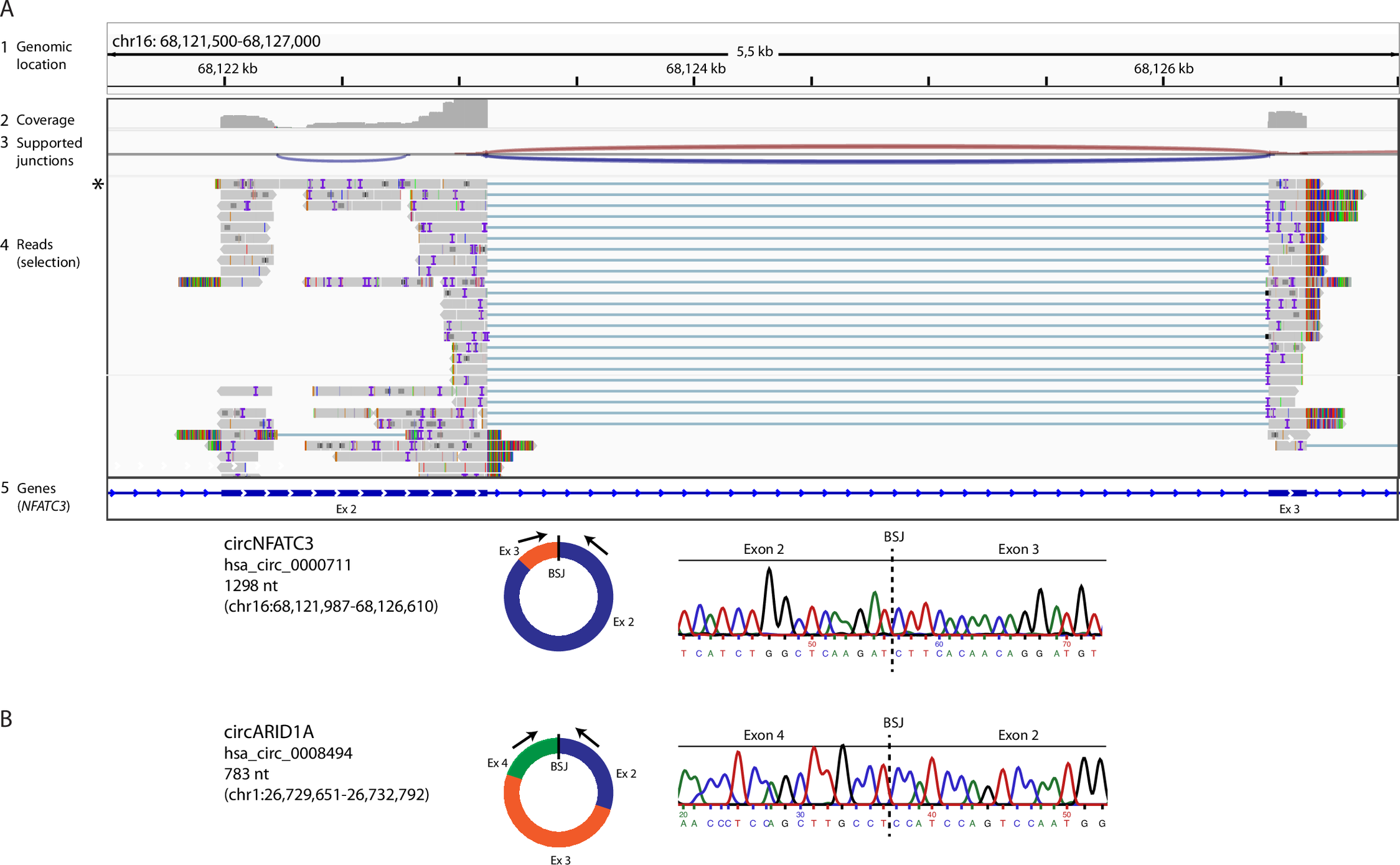

Supplement: S1 Fig — 2 circRNAs detected by Nanopore-seq were validated by Sanger sequencing similar as in Fig 5. Representative alignments and the BSJ-sequence obtained by Sanger sequencing are shown for A) circNFATC3 and B) circARID1A (alignments see Fig 4). The circbase.org ID is mentioned [14]. BSJ, back-splice junction. (TIF) [file pone.0273253.s001.tif]
