# Supplementary material for: Generation of full-length circular RNA libraries for Oxford Nanopore long-read sequencing
Source: PLoS One. 2022 Sep 7;17(9):e0273253. doi: 10.1371/journal.pone.0273253 (PMC9451095; doi:10.1371/journal.pone.0273253)
Supplement: S1 File — (PDF) [file pone.0273253.s002.pdf]

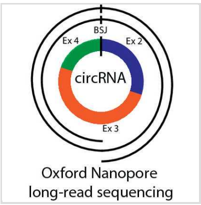

Version 2

Generation of full-length circRNA libraries for Oxford Nanopore long-read sequencing V.2

Steffen Fuchs[1], Loélia Babin[2], Elissa Andraos[2], Chloé Bessiere[2], Semjon Willier[3], Johannes H. Schulte[1], Christine Gaspin[4], Fabienne Meggetto[2]

[1]Charité - Universitätsmedizin Berlin, Germany;

[2]CRCT, Université de Toulouse, Inserm, CNRS, Université Toulouse III-Paul Sabatier, Centre de Recherches en Cancérologie de Toulouse, Toulouse, France;

[3]Dr. von Hauner Children's Hospital, University Hospital, LMU Munich, Germany;

[4]Université Fédérale de Toulouse, INRAE, BioinfOmics, GenoToul Bioinformatics facility, 31326, Castanet-Tolosan, France

dx.doi.org/10.17504/protocols.io.rm7vzy8r4lx1/v2

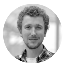

Steffen Fuchs
Charité - Universitätsmedizin Berlin, Université Toulouse II...

ABSTRACT

Circular RNA (circRNA) is a noncoding RNA class with broad implications for gene expression regulation, mostly by e.g. interaction with other RNAs or RNA-binding proteins. However, their specific sequence is not revealed by the commonly applied short-read Illumina sequencing. Here, we present an adapted protocol to enrich and sequence full-length circRNAs using the Oxford Nanopore long-read sequencing platform. The protocol involves an RNaseH-based ribodepletion, an enrichment of lowly abundant circRNAs by exonuclease treatment and negative selection of linear RNAs. Then, a cDNA library is created and amplified by PCR. This library is used as input for ligation-based sequencing together with native barcoding. Stringent quality control of the libraries is ensured by a combination of Qubit, Fragment Analyzer and qRT-PCR. The recommended amount of starting material is 7 µg of RNA, however lower amounts of RNA have also been tested successfully. Multiplexing of up to 4 libraries yields in total more than 1-2 Mio reads per library, of which 1-2 % are circRNA-specific reads with >99 % of them full-length. The protocol works well with human cancer cell lines. We further provide suggestions for Nanopore sequencing, the bioinformatic analysis of the created data, as well as the limitations of our approach and recommendations for troubleshooting and results interpretation. Taken together, this protocol enables reliable full-length analysis of circRNAs, a non-coding RNA type involved in a growing number of physiologic and pathologic conditions.

Steps

Enrichment of circRNAs for generation of Nanopore sequencing libraries

1. Ribodepletion
2. circRNA enrichment
3. cDNA library creation
4. Quality control

Suggestions for Nanopore sequencing and data analysis

5. Suggestions for Nanopore sequencing

6. Recommendations for bioinformatics analysis of the data
Expected results, limitations and troubleshooting
7. Expected results and interpretation
8. Limitations and challenges
9. Troubleshooting

The enrichment of the whole circRNA fraction follows the published workflow from Zhang *et al.* with several modifications:

- Modification of the ribodepletion method from a commercial kit to the published method of Baldwin *et al.*, which is based on a pool of DNA oligonucleotides that hybridize with ribosomal RNA and a digest of DNA:RNA hybrids by RNaseH

Baldwin A, Morris AR, Mukherjee N (2021). An Easy, Cost-Effective, and Scalable Method to Deplete Human Ribosomal RNA for RNA-seq.. Current protocols.
https://doi.org/10.1002/cpz1.176

- Clean-up steps and final size selection have been adapted to select for circRNAs longer than 200 nt and therefore also consider shorter circRNAs, whereas in the original protocol a selection of > 1kb was applied (the average length of circRNAs is between 200-800 nt)
- Additional negative poly(A) selection for further enrichment of circRNAs
- Increased quantity of retrieved library by using more starting material and a higher number of PCR cycles
- Thorough quality control by combining qRT-PCR, Qubit and Fragment Analyzer after circRNA enrichment

Further, we refer to the used sequencing protocol for the Nanopore platform, suggest changes to the standard protocol and further give recommendations for the bioinformatics analysis. We pool 4 libraries. The enrichment workflow can therefore be performed in parallel for 4 samples.

Zhang J, Hou L, Zuo Z, Ji P, Zhang X, Xue Y, Zhao F (2021). Comprehensive profiling of circular RNAs with nanopore sequencing and CIRI-long.. Nature biotechnology.
https://doi.org/10.1038/s41587-021-00842-6

DOI
dx.doi.org/10.17504/protocols.io.rm7vzy8r4lx1/v2

PROTOCOL CITATION

Steffen Fuchs, Loélia Babin, Elissa Andraos, Chloé Bessiere, Semjon Willier, Johannes H. Schulte, Christine Gaspin, Fabienne Meggetto . Generation of full-length circRNA libraries for Oxford Nanopore long-read sequencing. **protocols.io**
https://dx.doi.org/10.17504/protocols.io.rm7vzy8r4lx1/v2
Version created by Steffen Fuchs

KEYWORDS

Oxford Nanopore, circRNA, long-read sequencing, noncoding RNA, Transcriptomics

LICENSE

This is an open access protocol distributed under the terms of the Creative Commons Attribution License, which permits unrestricted use, distribution, and reproduction in any medium, provided the original author and source are credited

CREATED

Jun 22, 2022

LAST MODIFIED

Jun 24, 2022

PROTOCOL INTEGER ID

65089

GUIDELINES

General considerations:

- Work with RNA on ice whenever possible
- Prepare reactions in RNase-free reaction tubes, perform magnetic separations in 0.2 ml PCR stripes
- Perform incubation steps in a thermocycler
- When performing several reactions it is preferable to pipette a master mix including 10 % excess for pipetting errors
- Only use molecular grade nuclease-free H2O
- Clean-ups are performed with magnetic beads on a magnetic rack. Use a **ratio of beads and sample of 1:1** to select for RNAs > 150-200 nt, if not mentioned otherwise.

MATERIALS TEXT

General materials:

- RNase-free barrier tips, low retention
- 1.5 ml and 0.2 ml nuclease-free, low-retention microcentrifuge tubes, e.g. DNA LoBind tubes (Eppendorf, #0030108035)
- Thermocycler with thermal block suitable for 0.2 ml tubes, ability to ramp
- Tabletop microcentrifuge
- Magnetic tube rack for 0.2 ml strip tubes
- Ice bucket

- Nuclease-free water
- Thermocycler suitable for qRT-PCR, e.g. the Step One Plus Real Time PCR System (Applied Biosystems, RRID:SCR_015805)

For the ribodepletion:

- 7 µg of purified human total RNA in nuclease-free water, e.g. we used the following 4 anaplastic large-cell lymphoma (ALCL) cell lines as models: SU-DHL-1 (DSMZ Cat# ACC-356, RRID:CVCL_0538), Karpas-299 (DSMZ Cat# ACC-31, RRID:CVCL_1324), COST (RRID:CVCL_9491; Lamant *et al.*, 2004) and SUP-M2 (DSMZ Cat# ACC-509, RRID:CVCL_2209).

The protocol was also tested with lower amounts of RNA (3-5 µg), which did not affect the final number of reads, especially when multiplexing. However, 7 µg is the optimal amount for several rounds of sequencing.

Lamant L, Espinos E, Duplantier M, Dastugue N, Robert A, Allouche M, Ragab J, Brousset P, Villalva C, Gascoyne RD, Al Saati T, Delsol G (2004). Establishment of a novel anaplastic large-cell lymphoma-cell line (COST) from a 'small-cell variant' of ALCL.. Leukemia.

- Human rRNA depletion oligonucleotides, pooled at 200 µM in H2O

The 195 different 50mer oligonucleotides directed against human ribosomal RNA can be ordered e.g. from IDT (https://eu.idtdna.com) as lyophilized "oPool" oligo pool with a synthesis scale of 50 pmol/oligo. To reconstitute the oligos: spin down, resuspend the pool in **48.7 µL** H2O, incubate for **00:03:00** at **55 °C** . Continue incubating at **Room temperature** while smoothly shaking overnight. Separate in **10 µL** aliquots and store at **-20 °C** . For sequence information see supplementary table 1 of Baldwin *et al.*, 2021.

Baldwin A, Morris AR, Mukherjee N (2021). An Easy, Cost-Effective, and Scalable Method to Deplete Human Ribosomal RNA for RNA-seq.. Current protocols.
https://doi.org/10.1002/cpz1.176

- 5×RNase H buffer (250 mM Tris-HCl, pH 7.5, 500 mM NaCl, fill-up with nuclease-free H2O, store at 4°C)
- Agencourt RNA Clean XP beads (Beckman Coulter, #A63987)
- 100 mM MgCl2
- Hybridase Thermostable RNase H (Lucigen, # H39500)

- 1 mM EDTA
- Turbo DNase 2U/µl (Invitrogen, # AM2238) with 10x reaction buffer
- 80% (v/v) ethanol, molecular grade, made with nuclease-free water, freshly prepared

For the circRNA enrichment

- E. coli Poly(A) Polymerase (NEB# M0276) with 10x reaction buffer
- RNase inhibitor, murine (NEB #M0314S)
- RNaseR (Epicentre/Lucigen #RNR07250) with 10x reaction buffer
- NEBNext® Poly(A) mRNA Magnetic Isolation Module (NEB #E7490)

For the cDNA library generation

- SMARTer cDNA synthesis kit (Takara, #634926)
- Custom SMARTer CDS Primer II A. The primer of the kit is replaced by a custom DNA oligo, which can be ordered from any oligonucleotide provider, e.g. IDT. Sequence: 5'-AAGCAGTGGTATCAACGCAGAGTACNNNNNN-3', (N= A, C, G, T) dilute to a concentration of 12 uM.
- LongAmp Taq 2x Master Mix (NEB, #M0287S)
- Agencourt AMPure XP beads (Beckman Coulter, #A63880)

For the quality control

- Qubit 4 fluorometer (Thermo Fisher Scientific, RRID:SCR_018095) with kit Broad-range dsDNA (#Q33230)
- Fragment analyzer 5400 (Agilent, RRID:SCR_019410) with kit hsNGS Fragment (1-6000 bp, #DNF-474-1000)
- Maxima H Minus First Strand cDNA Synthesis Kit (Thermo Fisher Scientific #K1681)
- FastStart Essential DNA Green Master (Roche, #06402712001)
- Primer suitable for qRT-PCR to control for depletion of unwanted transcripts and enrichment of circRNAs (see table 1 below), 10 µM concentration. Please refer to the section "Quality control" for information about primer design.

For the quality control reverse transcription is performed with the Maxima H Minus First Strand cDNA Synthesis kit, since the SMARTer cDNA synthesis kit is relatively expensive.

Depending on your cell model choose abundant circRNAs as positive controls for the circRNA enrichment workflow. The here provided examples of circRNAs might not be expressed in your particular cell line.

Use divergent primers to specifically amplify circRNAs by qRT-PCR.

A	B	C	D
**Target**	**Primer**	**Sequence 5' - 3'**	**Comment, cirbase.org ID**
	SMARTer CDS Primer II A	AAGCAGTGGTATCAACGCAGAGTACNNNNNN	N is variable and can be A,C,G,T to amplify non-polyA transcripts. Design by Zhang, 2021, PMID 33707777
18S rRNA	18S_F	GTAACCCGTTGAACCCCATT	
	18S_R	CCATCCAATCGGTAGTAGCG	
MT-RNR1	MT-RNR1_F	AAACTGCTCGCCAGAACACT	
	MT-RNR1_R	GAGGTGGTGAGGTTGATCGG	
RN7SL2	RN7SL2_F	CTGTAGTGCGCTATGCCGA	
	RN7SL2_R	TGATCAGCACGGGAGTTTTGA	
RNU6B	RNU6B_F	CTCGCTTCGGCAGCACATATACTA	Design by Memczak, 2013, PMID 23446348
	RNU6B_R	ACGAATTTGCGTGTCATCCTTGCG	
HIPK3	circHIPK3_F	TATGTTGGTGGATCCTGTTCGGCA	Design by Zheng, 2016; PMID 27050392
	circHIPK3_R	TGGTGGGTAGACCAAGACTTGTGA	hsa_circ_0000284
	linHIPK3_F	AGTGCCAGAACAGGAGTAATTCA	Design by Zheng, 2016; PMID 27050392
	linHIPK3_R	CGGGGAGTCGGCAATAATGA	
ZBTB46	circZBTB46_F	CCGGTAGTGGGACGTGATTT	hsa_circ_0002805
	circZBTB46_R	ACTCGCTGTCCCAGTCTGTA	
ZKSCAN	circZKSCAN_F	CAGTCACGAGGAATAGTAAAGAAAC	hsa_circ_000127
	circZKSCAN_R	TCCAAACAGGGTCTGTGCTC	
	linZKSCAN1_F	CCGGGGCTCACGGAATAGTA	
	linZKSCAN1_R	GCTTCCCGTGATTCAGCAGT	

Table 1: Primers used for cDNA library creation and for the quality control qRT-PCR. The unique circbase.org ID is reported for each circRNA and the reference, if it was not an own design.

Glažar P, Papavasileiou P, Rajewsky N (2014). circBase: a database for circular RNAs.. RNA (New York, N.Y.).
https://doi.org/10.1261/rna.043687.113

SAFETY WARNINGS

Please refer to the safety warnings provided by the manufacturers of the individual reagents and chemicals. Ethanol is highly inflammable.

BEFORE STARTING

Work in an RNase-free workspace. Clean your workspace and tools with a product, such as RNase Zap (Invitrogen, #AM9780).

1) Ribodepletion 2h 30m

1 ***Enrichment of circRNAs for generation of Nanopore sequencing libraries***

This section provides the detailed protocol to enrich the fraction of circRNAs that will be used to create sequencing libraries for the Nanopore platform.

2 **Ribodepletion**

Ribodepletion will eliminate the majority of ribosomal RNAs (rRNA) that consist of > 80% of total RNA. This is an important step for the final enrichment of circRNAs. The ribodepletion follows the published protocol from Baldwin *et al.* 2021 with some modifications, which uses a pool of 195 DNA oligonucleotides directed against human rRNAs. DNA:RNA duplexes get digested by RNaseH and remaining DNA oligos will be digested by DNase. We multiplex usually 4 samples that can be treated in parallel throughout the workflow.

Baldwin A, Morris AR, Mukherjee N (2021). An Easy, Cost-Effective, and Scalable Method to Deplete Human Ribosomal RNA for RNA-seq.. Current protocols.
https://doi.org/10.1002/cpz1.176

It is recommended to also do an untreated Mock control through the protocol that will be used for the comparison of circRNA enrichment in the section quality control.

3 **Hybridization and RNaseH treatment**

Prepare the following: thaw **7 µg** total RNA **On ice**

The protocol was tried with lower amounts of RNA, but the amount of library obtained in the end seemed optimal with 7 µg of input.

RNA integrity should be assessed by methods such as e.g. Agilent Fragment Analyzer (Fig. 1). An RQN of >8.0 is preferable.

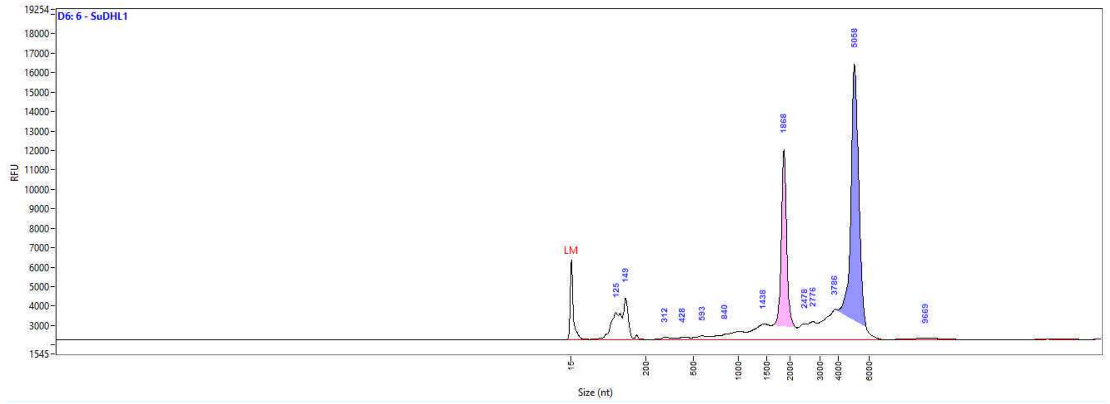

Figure 1: Example of an RNA sample with high RNA integrity. Isolated RNA of SU-DHL-1 cells was analyzed by Fragment Analyzer with the hs RNA kit. The 18S and 28S peaks are marked. The RQN of this sample is 9.6. RFU, relative fluorescence unit.

RNA quantity can be measured by methods such as Nanodrop or Qubit.

3.1 Thaw one aliquot of rRNA depletion oligos **On ice**

3.2 Bring one aliquot of Agencourt RNA Clean XP beads to **Room temperature** at least **00:30:00** before use. 30m

3.3 Set a heat block to **65 °C**

This will be used to warm up the *RNaseH mix*.

3.4 Set up the following PCR program in a thermocycler:

- 95°C - Hold (to heat up the thermocycler)
- 95°C - 3 min.
- Ramp: 0.1°C/s to 65°C
- 65°C - 5 min.
- 65°C - Hold (this is when the mixes will be put together)
- 65°C - 10 min.

3.5 Prepare the *RNaseH mix* and incubate it in the heat block (**step 3.3**) at 65°C **without adding the RNaseH enzyme**:

A	B	C
**Reagent**	**Volume per Reaction [µl]**	**Final concentration**
5x RNaseH buffer	4	1x
100 mM MgCl2	6	10 mM
5 U/µl RNaseH enyzme	4 (add later)	10 U
H2O	6	
**Total volume**	**20**	

The RNaseH enzyme is added to the *RNaseH mix* right before the mix is added to the hybridized samples.

3.6 Prepare the *hybridization mix*:

A	B	C
**Reagent**	**Volume per Reaction [µl]**	**Final concentration**
5x RNaseH buffer	8	1x
1 mM EDTA	2	50 µM
rRNA depletion oligos (200 µM)	9.9	30 µg
7 µg of total RNA	x	7 µg
H2O	fill up to 40 µl	
**Total volume**	**40 µl**	

If the amount of RNA is changed, keep a ratio of RNA:oligos of 1:4-5.

3.7 Incubate the sample for hybridization in the programmed thermocycler from **step 3.4** until the **65 °C** hold is reached.

3.8 Add the RNaseH enzyme to the *RNaseH mix* from **step 3.5**. Mix well by pipetting and spin down. Add **20 µL** of the *RNaseH mix* now with the RNaseH enzyme to the *hybridization mix* from **step 3.6**. Mix well by pipetting and spin down. Place back in the thermocycler.

3.9 Continue the program of the thermocycler (**65 °C** for **00:10:00**) then place the sample **On ice** 10m

4 First bead clean-up

Prepare the following:

This describes the general process of a bead-based sample clean-up that will be used in a similar way throughout the protocol. Usually a 1:1 ratio of sample volume to bead volume should be kept, if not otherwise stated to select for fragments > 150-200 nt.

4.1 Leave RNA Clean XP beads for **00:30:00** at **Room temperature** before using them. Vortex thoroughly the RNA Clean XP beads until the solution is homogeneously brown. 30m

4.2 Add 1x RNA Clean XP beads (**60 µL**) to the RNase-H treated sample and mix well by pipetting until the solution is homogenous.

4.3 Incubate for **00:05:00** at **Room temperature** 5m

4.4 Place sample on the magnet and incubate at **Room temperature** for at least **00:05:00** or until the beads are clearly separated from the supernatant. Discard supernatant without disturbing the beads. 5m

4.5 Perform **2 washes with freshly prepared ethanol 80 %** without disturbing the beads:

4.6 While leaving the sample on the magnet add **200 µL** Ethanol 80 % without disturbing the beads.

4.7 Incubate for **00:00:30** at **Room temperature** . Remove the supernatant without disturbing the beads. 30s

4.8 Repeat **steps 3.6** and **3.7**.

4.9 Let samples air-dry (leave lid open) for **00:05:00** , take care not to over-dry the beads (overdrying is indicated by formation of cracks in the bead pellet). 5m

4.10 Remove samples from the magnet, add **38.5 µL** H_2O to the beads, resuspend well by pipetting. Incubate **00:05:00** at **Room temperature** . 5m

4.11 Place the samples back on the magnet for **00:05:00** or at least until the beads are clearly separated from the supernatant. 5m

4.12 Transfer **37.5 µL** of the supernatant to a new 0.2 ml tube (try not to transfer the beads).

5 Digestion of ribodepletion oligos by DNase

Prepare the *DNase mix*:

A	B	C
**Reagent**	**Volume per reaction [µl]**	**Final concentration**
Turbo DNase (2U/µl)	12	24 U
10x DNase buffer	5.5	1x
**Total**	**17.5**	

30m

5.1 Add 17.5 µL of DNase mix to the cleaned-up sample from before, mix well by pipetting and incubate at 37 °C for 00:30:00 in a thermocycler.

5.2 Place On ice after incubation.

6 **Second bead clean-up**

Follow the procedure described for the first bead clean-up using 1x RNA Clean XP beads (55 µL).

6.1 Elute in 15.5 µL H2O.

6.2 Save 14.5 µL of supernatant for circRNA enrichment. Put On ice .

2) circRNA enrichment 2h 45m

7 **Polyadenylation of linear RNAs**

RNaseR is an exonuclease that will digest linear RNAs. It was shown that prior adenylation of linear RNAs by Poly(A) polymerase to extend their 3' extremities leads to more efficient depletion (Xiao 2019).

Xiao MS, Wilusz JE (2019). An improved method for circular RNA purification using RNase R that efficiently removes linear RNAs containing G-quadruplexes or structured 3' ends.. Nucleic acids research.
https://doi.org/10.1093/nar/gkz576

7.1 Prepare the *polyadenylation mix*.

A	B	C
**Reagent**	**Volume per reaction [µl]**	**Final concentration**
Ribodepeted RNA	14.5	
ATP (10 mM)	2	1 mM
Poly(A) polymerase (5 U/µl)	1	5 U
10x reaction buffer	2	1x
RNase-inhibitor (40 U/µl)	0.5	20 U
**Total volume**	**20**	

7.2 Mix well by pipetting and spin down. Incubate **00:30:00** at **37 °C** in a thermocycler. 30m

7.3 Clean-up with RNA Clean XP beads as described before, using a 1:1 ratio (**20 µL** beads).

7.4 Elute in **18 µL** H2O, save **17 µL** for RNaseR treatment. Put **On ice**

8 RNaseR treatment

RNaseR will degrade the now polyadenylated linear RNAs, whereas most circRNAs are resistant. This will enrich for circRNAs.

8.1 Prepare the *RNaseR mix*.

A	B	C
**Reagent**	**Volume per Rx [µl]**	**Final concentration**
Polyadenylated RNA	17	
10x reaction buffer	2	1x
RNaseR (20U/µl)	0.5	10u
RNase inhibitor (40u/µl)	0.5	20u
**Total volume**	**20**	

8.2 Incubate for 00:30:00 at 37 °C in a thermocycler. 30m

8.3 Clean-up with RNA Clean XP beads as described before, using a 1:1 ratio (20 µL beads). Elute in 21 µL , save 20 µL for negative poly-a selection. Put On ice .

9 Negative poly(A) selection

Oligo(dT) coupled beads will be used to negatively select the remaining polyadenylated linear RNAs. This step follows the manufacturer's protocol of the NEBNext® Poly(A) mRNA Magnetic Isolation Module (NEB #7490) with modifications. This further enriches for circRNAs.

9.1 Bring the NEBNext® Poly(A) mRNA Magnetic Isolation Module to Room temperature

9.2 Vortex oligo(dT) beads until the solution is homogenous.

9.3 Take 20 µL oligo(dT) beads in a separate PCR tube and wash twice with 100 µL of RNA bead binding buffer by using a magnetic rack, resuspend the beads in 20 µL of RNA bead binding buffer (1:1 ratio with RNA volume). Mix beads and RNA by pipetting.

9.4 Incubate 00:05:00 at 65 °C and hold at 4 °C in a thermocycler. 5m

9.5 Mix well by pipetting, leave 00:05:00 at Room temperature . 5m

9.6 Mix well by pipetting, leave 00:05:00 at Room temperature . 5m

9.7 Place tubes on the magnetic rack for 00:05:00 or until the liquid is clear. 5m

9.8 ⚠

Save the supernatant in a new PCR tube and keep On ice .

> The supernatant consists of the non-poly(A) fraction, including circRNAs. The poly(A) linear RNA is bound to the beads.

> ⚠ Don't discard the supernatant, which is the enriched circRNA fraction.

9.9 Clean-up with RNA Clean XP beads as described before, using a 1:1 ratio (40 µL).

9.10 Elute in 6 µL , save 5 µL for cDNA library creation and quality control, keep On ice .

3) cDNA library creation 3h 30m

10

> The enriched circRNA fraction will be used to create a cDNA library using the SMARTer cDNA synthesis kit (Takara #634926) following the manufacturer's instructions with modifications. To transcribe circRNAs without poly(A) sequences the 3'SMART CDS Primer IIA, which has a 3'oligo(dT) has to be replaced by a primer with random nucleotides at the 3' end. The cDNA library is amplified with LongAmp Taq 2x master mix (NEB # M0287S) using the SMARTer PCR primer from the cDNA synthesis kit.

11 **cDNA generation**

Set up the following PCR program in a thermocycler:

- 3 min. at 72°C
- 10 min. at 25°C
- Hold at 42°C (this is when the *reverse transcription mix* gets added)
- 90 min. at 42°C
- 10 min. at 70°C
- Hold at 4°C

11.1 ⚠

Prepare the *hybridization mix*:

A	B	C
**Reagent**	**Volume per Rx [µl]**	**Final concentration**
RNA	3.5	
Custom SMARTer CDS Primer IIA (12 µM)	1	2.7
**Total volume**	**4.5**	

Mix well by pipetting. Incubate in the programmed thermocycler until the **42 °C** hold is reached.

Save the remaining RNA from **step 9.10**. It will be used for the quality control later.

11.2 In the meantime prepare the *reverse transcription mix* and add it to the *hybridization mix*, when the **42 °C** hold is reached:

A	B	C
**Reagent**	**Volume per Rx [µl]**	**Final concentration**
5x First-strand buffer	2	0.36x
Dithiotreitol (DTT, 100 mM)	0.25	4.5 mM
dNTP mix (10 mM)	1	1.8 mM
SMARTer IIA oligo (12 µM)	1	2.2 µM
RNase inhbitor (40 U/µl)	0.25	10 U
SMARTScribe RT (100 U/µl)	1	100 U
**Total volume**	**5.5**	

Mix well by pipetting, continue the PCR program until the 4 °C hold is reached. Keep the sample On ice .

12 PCR amplification

Prepare the *PCR mix*:

A	B	C
**Reagent**	**Volume per Rx [µl]**	**Final concentration**
SMARTer PCR primer (12 uM)	6.8	0.8
cDNA	4	
LongAmp Taq 2x Master mix	50	1x
H20	39.2	
**Total**	**100**	

12.1

Incubate using the following PCR program

A	B	C
**Step**	**Temperature [°C]**	**Time**
Initial denaturation	95	30 s
25 cycles	95	15 s
	62	15 s
	65	2 min
Final extension	65	2 min
Hold	4	

13 Fragment size selection

Clean-up with **AMPure XP beads (DNA-specific)** as described before, **using a 0.8:1 ratio** (80 µL beads) to select for fragments > 200 nt. Elute in 31 µL H2O, save 30 µL for creation of libraries for Oxford Nanopore sequencing and quality control. Keep On ice .

13.1

The cDNA library can be stored at -20 °C for several weeks.

4) Quality control 5h 30m

14 Assessing the molarity of the library

Preparation of the library for Oxford Nanopore sequencing requires calculation of the molarity of the library, or in other words, the number of fragments that can be sequenced. Therefore, a quantification and also length determination of the library is necessary.

14.1 *Quantification of the library*

Quantify the library by using a Qubit fluorometer with the BR dsDNA assay, following the manufacturer's instructions and using **1 µL** of amplified cDNA library.

14.2 *Determination of the library size and quality*

Check the average length of the library by e.g. Fragment Analyzer (hs NGS Fragment kit, **2 µL** of diluted amplified cDNA library as input) by following the manufacturer's instructions.

14.3 *Calculation of the library's molarity*

Calculate the amount of fmol in your sample by using the results of the quantification and library size determination. This can be done by using e.g. the Biomath Calculator from Promega (Link to Promega Biomath calculatores) using the calculator DNA: µg to pmol. The recommended input for Oxford Nanopore library preparation with the kit SQK-LSK109 is 1 µg (or 100-200 fmol) of PCR amplicons. Since the circRNA abundance is still relatively low, multiplexing of several libraries with the native barcoding kit (EXP-NBD104) is recommended to occupy enough pores.

The input recommendation of the Oxford Nanopore library protocol EXP-NBD104 and SQK-LSK109 is 100-200 fmol for each library that will be pooled. In our experience, this can be increased up to 1000 fmol to have enough material to sequence lowly abundant circRNAs and do several rounds of sequencing.

15 Validation of circRNA enrichment

3h 30m

The enriched RNA fraction from **step 9.10** will be used to perform a qRT-PCR of the treated sample in comparison to a mock control to validate the enrichment of circRNAs and the depletion of unwanted RNA species. The following primers will be used that target:

Positve controls:

- 2-3 circRNAs of which you know that they are abundant in your cell line, e.g. by previous Illumina Total RNA-sequencing

Negative controls:

- Ribosomal RNA (rRNAs): 18s rRNA
- Mitochondrial RNA (mtRNAs): mtRNR1
- Small nucleolar RNA (snoRNA): RNU6B
- Signal recognition particle RNA (SRP): RN7SL2
- Linear RNAs, e.g. the cognate mRNAs of the selected circRNAs

This selection can be adapted to individual purposes.

Design of primers to specifically amplify circRNAs

To specifically amplify the backsplice-junction of circRNAs and not the cognate linear RNAs transcribed from the same gene, divergent (outward-facing) primer pairs have to be designed that will only lead to a PCR product if the template is circular. It is preferable to have one primer spanning the backsplice-junction to increase specificity, which is especially important when several isoforms of a circRNA exist. Common tools to design circRNA-specific primers are CircInteractome (Dudekula, 2016) or CircPrimer 2.0 (Zhong, 2022). Alternatively, NCBI Primer-BLAST can be used manually by taking the sequence of the backsplice-junction (ca. 100 nt upstream and downstream) and inverting the order of the exons forming the junction.

Dudekula DB, Panda AC, Grammatikakis I, De S, Abdelmohsen K, Gorospe M (2016). CircInteractome: A web tool for exploring circular RNAs and their interacting proteins and microRNAs.. RNA biology.
https://doi.org/10.1080/15476286.2015.1128065

Zhong S, Feng J (2022). CircPrimer 2.0: a software for annotating circRNAs and predicting translation potential of circRNAs.. BMC bioinformatics.
https://doi.org/10.1186/s12859-022-04705-y

15.1 Use 1 µL of enriched RNA from **step 9.10** as input for reverse transcription using the Maxima H Minus RT kit (Thermo #K1682) without DNase treatment.

15.2 Prepare the *reverse transcription mix*:

A	B	C
**Reagent**	**Volume per Rx [µl]**	**Final concentration**
Enriched RNA	1	
H2O	12.5	
Random hexamer primer (100 µM)	0.5	2.5 µM
dNTP Mix (10 mM)	1	0.5 mM
5X RT Buffer	4	1x
Maxima H Minus enzyme mix	1	
**Total volume**	**20**	

15.3 Mix by pipetting. Program a thermocycler and incubate the sample as follows:

- 10 min at 25 °C
- 15 min at 50 °C
- 5min at 85°C
- Hold at 4°C

15.4 Dilute 1 µL of cDNA 1:10 and use it as input for qRT-PCR using the FastStart Essential DNA Green Master 2x (Roche #06402712001).

15.5

Prepare the *qRT-PCR mix* for each target RNA:

A	B	C
**Reagent**	**Volume per Rx [µl]**	**Final concentration**
FastStart Essential DNA Green Master 2x	5	1x
Forward primer [10 µM]	0.5	0.5 µM
Reverse primer [10 µM]	0.5	0.5 µM
H20	3	
cDNA	1	
**Total volume**	**10**	

Preparation of a master mix for several samples is recommended.

15.6 Run for 40 cycles with the following PCR program:

A	B	C
**Step**	**Temperature [°C]**	**Time**
	50	2 min
	95	10 min
40 cycles	95	15 s
	60	60 s
Run melt curve analysis		

Analyze the qRT-PCR by comparing the expression of the RNA targets in the enriched sample with the Mock control.

5) Suggestions for Nanopore sequencing

16 ***Suggestions for Nanopore sequencing and data analysis***

This section provides links to the used Nanopore sequencing protocols with further information and improvements, and suggestions for the bioinformatics analysis.

17 **Protocols for Oxford Nanopore sequencing:**

Protocols are available from the Nanopore community (free login required):
The following protocol was used according to the manufacturer:

[Native barcoding amplicons (with EXP-NBD104, EXP-NBD114, and SQK-LSK109) v12Nov2019]

We included small modifications that we will describe below together with the general steps of the library preparation. We use an Oxford Nanopore MinION MK1C sequencer that includes a graphical card needed for base calling of the raw Nanopore sequencing data (.fast5 format) to generate .fastq files that are needed for further analysis. A standard MinION can be used as well and base calling can be performed afterwards with a computer and the Nanopore MinKNOW software.

17.1 *1) End-preparation:*

This step prepares the DNA ends for adapter attachment. The manufacturer recommends 100-200 fmol of RNA input. We use up to 1000 fmol, to have enough material for several rounds of sequencing. Our circRNA-enrichment workflow provides enough output to achieve this.

17.2 *2) Native barcode ligation and pooling:*

Nanopore barcodes will be attached to the ends of the DNA in this step. The manufacturer recommends to use 100-200 fmol of end-prepped DNA. Here, we used 22.5 µl of the reaction from step 1). The samples are cleaned-up with magnetic beads and eluted. The manufacturer recommends a high elution volume, which would make a concentration step necessary whenmultiplexing various samples. To avoid this, we elute only in 11 µl, to obtain 10 µl of sample for the pooling and 1 µl to measure the concentration (see below).

For the pooling, we measure the concentration of the sampleswith a Qubit fluorometer (BR dsDNA assay) and calculate the molarity as described above. Expected recovery aim 15-25 ng/µl (molarity 30-70 fmol/µl). Equimolar amounts of samples should be pooled. The protocol recommends 100-200 fmol, we usually use 400 fmol per sample and pool 4-5 samples.

17.3 *3) Adapter ligation and clean-up*

This step attaches the Nanopore sequencing adapters. After adding the adapters a clean-up is performed with magnetic beads. The manufacturer uses a ratio of 0.5x beads to sample to select for long fragments >500 nt. However, we use a ratio of 0.8x to include as well fragments of >200nt, since the expected size of circRNAs is 200-800 nt (Guo, 2014; Zheng, 2019). This also is the reason why the short fragment buffer has to be used for the clean-up. We perform in total 3 washes with the short fragment buffer to eliminate chemical components that might potentially interfere with sequencing (only 2 are recommended in the official protocol). The cleaned-up sequencing pool is measured with a Qubit fluorometer (BR dsDNA assay). Recovery aim: 30-50 ng/µl (ca. 50-90 fmol/µl). To calculate the molarity, we use the average length (as measured in the QC section by Fragment Analyzer) of all samples pooled. The final library pool is ready for sequencing and should be stored for short-term at 4 °C until the sequencing run is finished for possible flushing of the flow cell and reloading of the library (see below).

17.4 *4) Priming and loading the flow cell*

Finally the flow cell is primed and the library loaded. Please note that the pores in the flow cell needed for sequencing are not very stable and the number of active pores correlates with the sequencing output. Care has to be taken to keep as much pores active for sequencing as possible. For instance, air bubbles that get introduced by pipetting during the priming process can irreversibly damage them. Further, during priming the storage buffer that keeps the pores stable is taken off. The manufacturer recommends to wait 5 min. until the library gets loaded. In our experience it is best to prepare the library mix before starting the priming process so that the pores are as short as possible without the storage buffer.
The recommendation of the manufacturer is to load 5-50 fmol. In our experience this can be increased to 200 fmol. Of note, this does still not fully saturate the pores.
Sequencing is started on the MK1C with the base calling option activated using standard settings. We further activate also demultiplexing (standard settings), to directly separate the reads in the respective samples.

> *Optional: washing the flow cell and reloading the library during the sequencing run*
>
> The pores get inactive over time, which is a normal process. However, when sequencing short fragments this process is much faster. We observe after 1d a significant number of inactive pores (unavailable state in the MinKNOW view). We recommend to perform then a nuclease flush of the flow cell using the Flow Cell Wash Kit (EXP-WSH004, Nanopore community login needed) according to the manufacturer. This will take off the library and helps to recover pores, in our experience around 10-20 %. We then perform another flow cell priming as described before und re-load the library pool (200-300 fmol). With this approach the usually obtained 8-10 million reads can be increased to up to 15 million reads.

6) Recommendations for bioinformatics analysis of the data

18 Bioinformatics analysis

Demultiplexing and base calling of raw data were realized by using the Guppy toolkit from the MinKNOW software (v22.05.8) using standard parameters as described above. This creates .fastq files from the raw .fast5 data. Only passed .fastq files assigned to samples were used for next analyses.

Analysis of the base-called sequencing data in fastq format involves the following steps:

• Cleaning the reads from the adapters with cutadapt v3.4

• Identifying circRNAs by using the CIRI-Long tool v1.0.3
• Creating alignments with minimap2 v2.19 and visualize circRNA-spanning reads in IGV Genomics Viewer v2.9.4.
The used genome annotation was GRCh38.

18.1 *Cleaning the reads*

Cleaning the reads was done with cutadapt v3.4 and removes the adapter sequences. The following settings were used for the analysis:
- Select reads with non-zero length (-m 1) and set less than 20 % error rate (-e 0.2)
- Command lines to clean the reads with the used adapter sequences:
cutadapt -e 0.2 -m 1 -g AAGCAGTGGTATCAACGCAGAGTAC -o FileName_5pTrimmed.fastq.gz FileName.fastq.gz
cutadapt -e 0.2 -m 1 -a GTACTCTGCGTTGATACCACTGCTT -o TrimmedFileName.fastq.gz FileName_5pTrimmed.fastq.gz

MARTIN, Marcel. Cutadapt removes adapter sequences from high-throughput sequencing reads. **EMBnet.journal**, [S.l.], v. 17, n. 1, p. pp. 10-12, may 2011. ISSN 2226-6089. Available at: <https://journal.embnet.org/index.php/embnetjournal/article/view/200>. doi:https://doi.org/10.14806/ej.17.1.200

18.2 *Identifying circRNAs*

We used the CIRI-long software (v1.0.3; Zhang, 2021) with default parameters to identify circRNAs. CIRI-long first splits reads produced by rolling circle amplification into repetitive fragments by searching for identical repeated sequences. This first step allows to detect the boundaries of circRNAs which are then aligned against the genome to generate a consensus sequence. It uses splice sites from known exon annotations and canonical de novo GT/AG splice signals to align junction sites but also non-canonical splice signals when canonical signals are not present. Of note, a bwa index is required for the reference genome.
Only passed reads, not considered unclassified, were used for the analysis.
- Command lines used:
CIRI-long call -i FileName.fastq.gz -o step1 -r genome.fa -p SampleName -a genome.gtf -t 10
CIRI-long collapse -i SampleFile.lst -o step2 -p SampleName -r genome.fa -a genome.gtf -t 10

CIRI-long will create several output files with the distinct circRNA isoforms detected and the number of reads in the different samples.
In step1, it provides fasta files of reads predicted as circRNA with related information on, for example, the size and genomic coordinates of the circRNA, and the identified splice signal. Isoforms from all samples are collapsed in

step2. The main outputs are an expression matrix providing collapsed isoforms counting and a gtf-like file that contains detailed information of circRNAs and annotation of circRNA back-spliced regions.

Zhang J, Hou L, Zuo Z, Ji P, Zhang X, Xue Y, Zhao F (2021). Comprehensive profiling of circular RNAs with nanopore sequencing and CIRI-long.. Nature biotechnology.
https://doi.org/10.1038/s41587-021-00842-6

18.3 *Alignments to visualize circRNA-spanning reads*

CIRI-Long involves an alignment step, but the bam files containing the aligned reads are not conserved. Therefore, we created separate alignments, by aligning long reads against the human genome (GRCh38) using the minimap2 software (v2.19, Li, 2018), converted in bam format by using samtools (v1.12, Li, 2009) and visualized using IGV Genomics Viewer (v2.9.4, Robinson, 2011) keeping supplementary alignments, which allows to visualize inverted segments.
- Command lines used:
minimap2 -ax splice -t 20 -uf -k14 genome_index FileName.fastq.gz > FileName.sam ; samtools view -b FileName.sam|samtools sort > FileName.bam ; samtools index FileName.bam).

Li H (2018). Minimap2: pairwise alignment for nucleotide sequences.. Bioinformatics (Oxford, England).
https://doi.org/10.1093/bioinformatics/bty191

Li H, Handsaker B, Wysoker A, Fennell T, Ruan J, Homer N, Marth G, Abecasis G, Durbin R, 1000 Genome Project Data Processing Subgroup. (2009). The Sequence Alignment/Map format and SAMtools.. Bioinformatics (Oxford, England).
https://doi.org/10.1093/bioinformatics/btp352

Robinson JT, Thorvaldsdóttir H, Winckler W, Guttman M, Lander ES, Getz G, Mesirov JP (2011). Integrative genomics viewer.. Nature biotechnology.
https://doi.org/10.1038/nbt.1754

19 *Expected Results, limitations and troubleshooting*

Expected results and interpretation

Using our protocol libraries of an average length of 606.75 nt and a concentration of 5 ng/µl, thus almost 150 ng in total can be generated (Fig. 2, Tab. 2). This will be enough for several rounds of sequencing, if a high number of reads is needed to also detect lower expressed circRNAs. The library length is about the average published size of circRNAs, which is reported to be between 200 - 800 nt (Guo, 2014; Zheng, 2019) and shows that our workflow does not fragment RNA and maintains its size.

Guo JU, Agarwal V, Guo H, Bartel DP (2014). Expanded identification and characterization of mammalian circular RNAs.. Genome biology.
https://doi.org/10.1186/s13059-014-0409-z

Zheng Y, Ji P, Chen S, Hou L, Zhao F (2019). Reconstruction of full-length circular RNAs enables isoform-level quantification.. Genome medicine.
https://doi.org/10.1186/s13073-019-0614-1

A	B	C	D	E	F
	**SU-DHL-1**	**Karpas-299**	**COST**	**SUP-M2**	**Average**
**Concentration [ng/µl]**	5.12	4.68	4.92	4.5	4.81
**Size [nt]**	654	629	573	571	606.8

Table 2: Results of the library preparation. Libraries for Nanopore sequencing were prepared of 4 different anaplastic large-cell lymphoma cell lines (SU-DHL1, Karpas-299, COST, SUP-M2).The concentration was measured with the Qubit BR dsDNA kit and library size by Fragment Analyzer with the hs NGS kit.

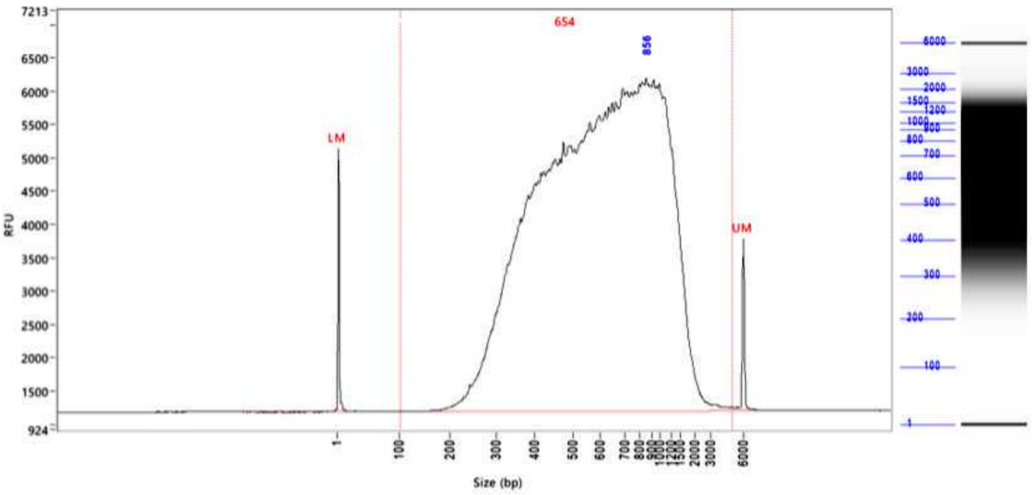

Figure 2: Generated libraries have the size of the average circRNA length. Shown is the library created from RNA of the anaplastic large-cell lymphoma cell lineSU-DHL-1. The library size was analyzed by Fragment Analyzer with the kit hs NGS. The average library size was 654 nt. RFU, relative fluorescence units.

Unwanted RNA transcripts should get depleted and the depletion is tested by qRT-PCR. Ribosomal RNA, as indicated by 18S rRNA, gets usually depleted more than 5 Cts, we achieve regularly a depletion of around 10 Ct. Further, mitochondrial RNA (as indicated by mtRNR1), small-nucleolar RNA (as indicated by RNU6B) and the RNA component of the signal recognition particle (as indicated by RN7SL2), which can be very abundant, should be depleted > 5 cycles. circRNAs should be stable or enriched, and cognate linear RNAs should get depleted (Fig. 3).

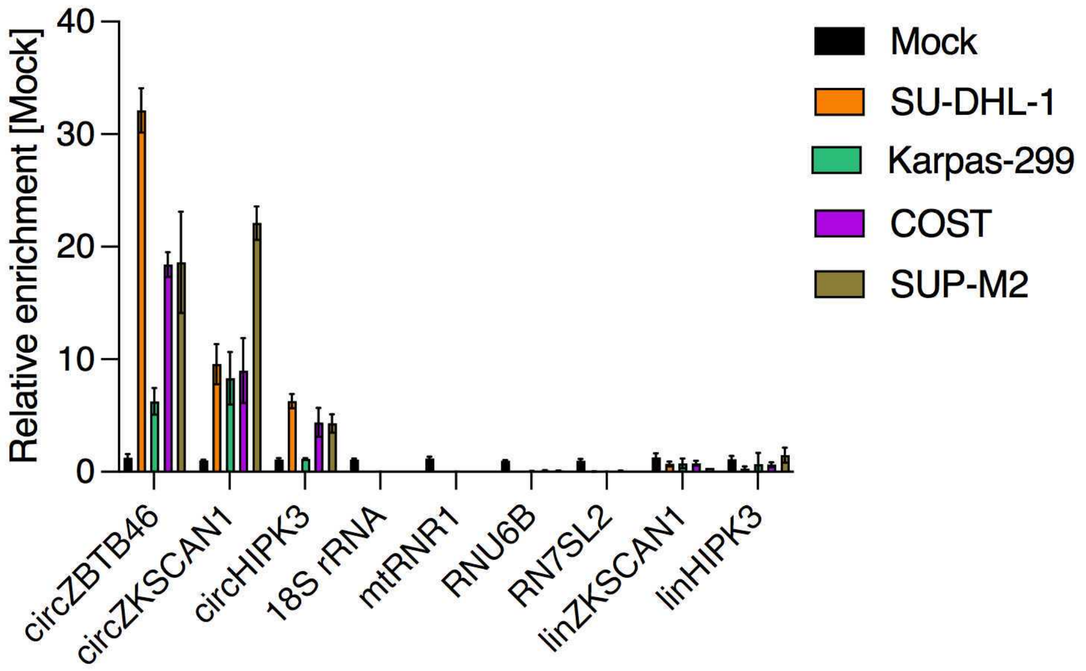

Figure 3: circRNAs get enriched by the library workflow. 4 different RNA samples from anaplastic large-cell lymphoma cell lines (SU-DHL-1, Karpas-299, COST and SUP-M2) were treated enzymatically to enrich for circRNAs as described in the protocol. The expression of circRNAs and unwanted transcripts (ribosomal RNA, 18S rRNA; mitochondrial RNA, mtRNR1; small-nucleolar RNA, RNU6B; signal recognition particle RNA, RN7SL2 and linear RNAs/mRNAs, linkZKSCAN1, linHIPK3) was analyzed by qRT-PCR and compared with an untreated Mock control.

Using the ligation-based sequencing kit (SQK-LSK109) together with the native barcoding kit (EXP-NBD104) 4 libraries of 4 different human anaplastic large-cell lymphoma cell lines (SU-DHL-1, Karpas-299, COST, SUP-M2) were pooled together and the pool was sequenced on a MinION (MK1C, Oxford Nanopore). The sequencing output was on average 1,536,242 reads per library and reads were of high quality (Tab. 3, mean Q-score 15).

A	B	C	D	E	F
	**SU-DHL1**	**Karpas-299**	**COST**	**SUP-M2**	**Average**
**Raw reads**	1,473,419	1,734,196	899,725	2,037,577	1,536,229
**Mean read length [nt]**	459.6	368.2	386.3	403.3	404.4
**Maximum read length [nt]**	4,006	3,889	3,538	3,455	3,722
**BSJ-reads [% of reads]**	1.05	0.95	0.95	1.06	1.00
**Full-length circRNAs**	15,673	16,725	8,750	21,918	15,767
**Different circRNAs**	3,143	3,195	1,426	4,017	2,945
**Mean circRNA length [nt]**	435.1	354.9	366.4	370.4	381.7
**Maximum circRNA length [nt]**	1,798	1,634	1,596	2,228	1,814

Table 3: Sequencing results obtained with one MinION flow cell. circRNA-enriched libraries from 4 anaplastic large-cell lymphoma cell lines were sequenced by Oxford Nanopore. Calculations are based on the passed reads and the circRNA analysis was performed with CIRI-Long.

The run took on average 40 h. Of note, the pores were not completely saturated, so probably a longer sequencing run with higher output would have been possible. Following the analysis

workflow described for CIRI-Long we could identify on average 15,767 circRNA-specific reads, thus 1.0 % of the total reads, similar to the study from Zhang *et al.*, of which 99 % covered the full length of the circRNA. For most of them, concatamers were detected and, as expected, several isoforms were identified and reported by CIRI-Long. On average 2,945 different circRNAs were identified. We noticed that the more reads are generated, more different full-length circRNA isoforms are detected, which could be another argument for deeper sequencing. The results were comparable among the samples from the 4 different cell lines, showing the robustness of the workflow.

In summary, this modified protocol facilitates consistent full-length sequencing of circRNAs, which will help to study this noncoding RNA type in a variety of physiologic and pathologic contexts.

8) Limitations and challenges

20 Limitations and challenges

A limitation of the protocol is the relatively high input of RNA of 7 µg. While this amount of RNA worked best in our experiments, we also tried successfully only 3-5 µg. Further, in general the Nanopore sequencing platform produces less reads than Illumina-based short read techniques, especially when using the MinION. That means lowly abundant circRNAs might not be detected by our sequencing protocol.

Limitations of CIRI-Long are that the alignment parameters cannot be modified and bam files containing the aligned reads are not conserved. Further, there is no option to detect fusion circRNAs derived from fusion genes (distant genes, genes located on different chromosomes).

9) Troubleshooting

21 Troubleshooting

Below we provide assistance and recommendations for problems that can occur during this protocol and the analysis of the data.

21.1 *circRNA enrichment*

- Step 7 RNaseR treatment: if the enrichment of circRNAs seems to be insufficient, a longer incubation with RNaseR could be tried.
- Step 11 PCR amplification: if the obtained amount of cDNA is much lower than expected, the volume of the PCR reaction and/or the amount of PCR cycles could be increased.
- Step 12 Fragment size selection: if the size of the circRNAs of interest that should be enriched is much higher or lower, the ratio of beads to DNA could be adapted. A higher ratio will lead to retention of smaller fragments, a lower ratio will select for longer fragments.

21.2 *Validation of circRNA enrichment*

- While we provide a selection of primers covering different RNA species that should be depleted by the workflow, further transcripts can be checked by qRT-PCR in this step.

21.3 *Nanopore sequencing*

- Care has to be taken to not introduce air bubbles while priming or loading a flow cell, since this will damage pores irreversibly.
- The pore activity should be closely monitored while sequencing. If a lot of inactive pores accumulate, a flow cell wash could be performed as described above.
- If a lot of pores are available, but not occupied, more library could be loaded. If the translocation speed is > 300, then no further priming is usually needed.

21.4 *Bioinformatics analysis*

- Alignments were re-generated using minimap2 (v2.19, GRCh38) and visualized in IGV Genomics Viewer (v2.9.4) to validate the presence of concatamers. Linking supplementary alignments also helped to visualize circularization junctions.
- To detect fusion circRNAs sam files produced by minimap2 during the alignment can be filtered to conserve chimeric alignments that contain segments of the same read aligning to distant genes (on the same or different chromosomes).
